# Predictors and Correlates of Prehospital Delay Among Acute Stroke Patients in Thiruvananthapuram District, Kerala: A Cross-Sectional Survey

**DOI:** 10.7759/cureus.79900

**Published:** 2025-03-01

**Authors:** Anjali Krishnan, Angelina Roy, Jithesh Vettilakath, Anjaly NT, Arun Babu

**Affiliations:** 1 Research, State Health Systems Resource Centre - Kerala, Thiruvananthapuram, IND

**Keywords:** acute stroke care, early arrival, functional outcomes, pre-hospital delay, therapeutic window period

## Abstract

Introduction

Stroke is a significant global public health challenge, contributing to high mortality rates and long-term disability. Beyond its physiological impact, stroke imposes a substantial socioeconomic burden on patients, families, and healthcare systems. Timely intervention, particularly through the prompt administration of reperfusion therapies, is crucial in reducing adverse outcomes. However, delays in reaching a healthcare facility after symptom onset often prevent patients from receiving these lifesaving treatments. Understanding the prevalence and factors contributing to prehospital delays is essential for improving stroke care and patient outcomes. This study aimed to assess the prevalence of prehospital delays and their correlates among stroke patients in Thiruvananthapuram district, Kerala, while also exploring the situational challenges patients face in accessing healthcare facilities.

Materials and methods

A hospital-based cross-sectional survey was conducted among 170 patients with confirmed acute stroke who presented to the emergency departments of four hospitals in Thiruvananthapuram district. Patients arriving within four and a half hours of symptom onset were classified as “early arrivals,” while those arriving after this period were categorized as “delayed/late arrivals.” Data collection was facilitated using Open Data Kit software and analyzed with IBM SPSS Statistics for Windows, Version 22.0 (Released 2013; IBM Corp., Armonk, NY, USA). Univariate and multivariate analyses were performed to identify associations. Additionally, freewheeling interviews were coded to complement and validate the quantitative findings.

Results

The median time from symptom onset to hospital arrival was 6.75 hours (IQR: 2.27-17.48 hours), with 40% of stroke patients experiencing prehospital delays. Bivariate analysis revealed significant associations between delay and factors such as age, housing type, income source, occupation, socioeconomic status, presence of dependents, choice of healthcare facility, number of facilities approached, and Modified Rankin Scale score. Multiple regression analysis identified housing type and the number of facilities approached as significant predictors of prehospital delay. Freewheeling interviews further indicated that, regardless of sociodemographic characteristics, hesitation, reluctance, and self-assessment were the primary reasons for delayed hospital arrival.

Conclusions

The study identified a high prevalence of prehospital delay (40%) among acute stroke patients in Thiruvananthapuram, the capital city of Kerala. These findings emphasize the need for health promotion strategies aimed at increasing public awareness of early stroke symptoms, ensuring the direct transfer of patients to hospitals equipped with CT scan and thrombolysis facilities, and standardizing referral processes with uniform protocols to minimize delays and improve patient outcomes.

## Introduction

Stroke is the second leading cause of mortality and the third leading cause of disability worldwide, with its impact being particularly severe in low- and middle-income countries [1]. Beyond individual health consequences, stroke imposes a significant socioeconomic burden on patients and their families, often resulting in long-term disability that affects physical, cognitive, and emotional well-being, reduces quality of life, lowers productivity, and increases healthcare costs [2,3].

In India, the burden of stroke is rising, making it the fourth leading cause of death and the fifth leading cause of disability [4]. Even in Kerala, a state with relatively strong health indicators, stroke incidence remains high [5], with an average annual rate of 145 cases per 100,000 people [6]. Jeffrey Saver’s concept of “time is brain” highlights the time-sensitive nature of stroke management, emphasizing the need for early medical intervention to minimize brain damage and improve survival rates and functional outcomes [7]. The optimal therapeutic window is within four and a half hours, with the best results achieved when treatment is initiated within 90 minutes [8]. The World Stroke Organization, the American Heart Association (AHA), and the Ministry of Health and Family Welfare, Government of India, have all issued recommendations stressing the importance of timely diagnosis and intervention [9]. However, in developing countries like India, severe delays in hospital presentation mean that only a small percentage of acute stroke patients receive thrombolysis, leading to its underutilization. These delays are largely due to systemic challenges, including prehospital delays [10].

Prehospital delay refers to the time between symptom onset and the initiation of treatment, which significantly affects patient outcomes. Therefore, minimizing this delay is crucial for improving stroke survival rates and recovery [11]. However, there is a lack of studies in India, particularly in South India, assessing the prevalence and determinants of prehospital delay [12,13]. This study aims to evaluate prehospital delay and its associated factors among acute stroke patients in Thiruvananthapuram district, Kerala. The findings could help develop strategies to reduce delays and enhance healthcare service delivery for stroke patients in the region. This research is particularly relevant in the context of Kerala’s expansion of the Stroke Identification, Rehabilitation Awareness, and Stabilization (SIRAS) initiative - a program designed to provide timely, cost-free stroke care through primary stroke care units [14].

## Materials and methods

A cross-sectional survey was conducted between July 2023 and April 2024 among acute stroke patients admitted to four selected hospitals in Thiruvananthapuram district, Kerala, who provided consent to participate. The sample size was determined using OpenEpi Version 3.0, based on a reported prevalence of prehospital delays of approximately 73.3% from an Indian study [11], with an absolute precision of 10% and a design effect of 2. The calculated sample size was 151 at a 95% CI. To account for a 10% nonresponse rate, the final sample size was rounded to 170.

Initially, a list of patients admitted during the study period was obtained from each hospital, and gatekeeper consent was secured. Deceased patients and those who did not respond after three consecutive phone calls were excluded. The principal and co-investigators then visited consenting patients at their residences based on their convenience. Eligible participants were adults aged 18 years or older who had been diagnosed with ischemic or hemorrhagic stroke using CT or MRI. Exclusion criteria included pregnant women, individuals with cognitive impairments, and those unable to respond to the questionnaire due to aphasia.

Before the interview, informed consent was obtained from all participants. In a small subset of cases (n = 12), stroke patients were unable to recall events leading up to hospital admission. In these instances, caregivers who had been present during the admission process were contacted to verify details of symptom onset and hospital arrival.

The survey targeted patients who could recall and confirm their hospital admission details, with caregivers assisting in cases where memory loss was a barrier. A structured questionnaire developed by the research team was used to collect data across four sections: (1) sociodemographic characteristics (age, gender, education, marital status, occupation, income source, and socioeconomic status); (2) household details (household size and type); (3) clinical details (comorbidities, stroke type, symptom onset time, and time of hospital arrival); and (4) functional outcomes (mRS (Modified Rankin Scale) and Health Quality Scale). Comorbidities were self-reported and validated using discharge summaries and the list of medications the patient was taking at the time of data collection.

Following the cross-sectional survey, 15 freewheeling interviews were conducted with selected patients or caregivers who had been present during admission. These participants were chosen purposively based on the initial quantitative survey. The interviews were transcribed, translated, and coded to complement the survey findings. During these interviews, additional insights were gathered on symptom recognition and the decision-making process for hospital transportation.

Ethical considerations

This study was conducted as part of a larger research project titled “Estimation of Catastrophic Health Expenditure and Its Coping Strategies Among Stroke Survivors in Thiruvananthapuram District, Kerala.” The study received approval from the Institutional Ethics Committee of SHSRC-Kerala (EC/NEW/IND/2022/2909). As per the National Ethical Guidelines of the Indian Council of Medical Research, it was classified as less than minimal risk. Privacy and confidentiality were strictly maintained throughout the study.

Operational definition

Prehospital delay was defined as the time interval from symptom onset to hospital arrival exceeding four and a half hours, where essential stroke care services were first received. This definition aligns with the AHA’s guidelines, which recommend administering recombinant tissue plasminogen activator within three to four and a half hours of stroke onset for eligible patients (Class I Recommendation, Level of Evidence B). It is also based on the study by Aref et al. [15,16].

Data analysis

The data was entered using Open Data Kit and exported to IBM SPSS Statistics for Windows, Version 22.0 (Released 2013; IBM Corp., Armonk, NY, USA)for analysis. Univariate analysis was performed for descriptive purposes, followed by coding and recoding of variables for bivariate analysis. Bivariate analysis was conducted using chi-square and Mann-Whitney U tests. For multivariate analysis, logistic regression was performed using the variables that showed significant associations with prehospital delay in the bivariate analysis. A p-value of less than 0.05 was considered statistically significant in both bivariate and multivariate analyses. Additionally, the freewheeling interviews were transcribed, translated, and coded to support the quantitative findings.

## Results

Profile of study participants

The sociodemographic and household details are presented in Table 1. The median age of the study participants was 66 years (IQR: 59.7-74.25), with a predominance of males (n = 113, 66.5%). Most participants had completed at least a high school education (n = 162, 94.8%) and resided in rural areas (n = 127, 74.7%). According to the Antyodaya Anna Yojana scheme classification by the Government of India, the majority belonged to the above poverty line (APL) category (n = 106, 62.3%). Approximately 72% (n = 123) reported having a monthly income, with the primary sources being an employee pension (28.5%), salary (26.4%), old-age pension (16.2%) (not regular), and gifts/assistance (1.2%). The median monthly income was reported as 5,000 INR (IQR: 0-20,000 INR). The median household size was four (IQR: 3-5), and household and treatment expenses were primarily managed by family members, particularly children, who held decision-making authority (n = 89, 53%).

**Table 1:** Profile of study participants APL, above poverty line; BPL, below poverty line; mRS, Modified Rankin Scale

Variable	Categories	Frequency, N (%)
Age in years	18-40	6 (3.5%)
	41-60	48 (28.2%)
	>60	116 (68.2%)
Gender	Male	113 (66.5%)
	Female	57 (33.5%)
Type of residence	Rural	127 (74.7%)
	Urban	43 (25.3%)
Marital status	Unmarried	6 (3.5%)
	Married and living with spouse	136 (80.0%)
	Widowed	27 (15.9%)
	Separated	1 (0.6%)
Education	No formal education	8 (4.7%)
	Up to high school	113 (66.5%)
	Higher secondary	14 (8.2%)
	Diploma	6 (3.5%)
	Graduation or above	29 (17.1%)
Occupation	Employed	96 (56.5%)
	Unemployed	74 (43.5%)
Economic status	Most economically backward	9 (5.3%)
	BPL	55 (32.4%)
	APL	106 (62.3%)
Patient’s income	0-5,000 INR	86 (50.6%)
	5,001-10,000 INR	16 (9.4%)
	10,001-30,000 INR	52 (30.6%)
	Above 30,000 INR	16 (9.4%)
Household head	Patient	132 (77.6%)
	Children	24 (14.2%)
	Others	14 (8.2%)
Household size	One to four members	102 (60%)
	Five to six members	56 (32.9%)
	More than six members	12 (7.1%)
Multiple comorbidities	Yes	109 (64.4%)
	No	60 (35.5%)
Type of comorbidities	Diabetes mellitus	90 (52.9%)
	Hypertension	118 (69.4%)
	Dyslipidemia	71 (41.8%)
	Cardiac disease	35 (20.6%)
	Respiratory disease	19 (11.2%)
	Musculoskeletal disorders	24 (14.1%)
	Others	20 (11.8%)
Number of facilities opted for stroke care	Single	58 (33.9%)
	Multiple	112 (65.5%)
mRs score at admission	Good outcome (1-2)	28 (16.5%)
	Bad outcome (3-5)	142 (83.5%)
mRs at present	Good outcome	105 (61.8%)
	Bad outcome	63 (37.1%)

Details of comorbidities and stroke-related factors are also summarized in Table 1. Substance addiction was observed in a small percentage of participants (5.8%), as most had quit alcohol and smoking after experiencing a stroke. Many patients (n = 112, 65.8%) visited multiple healthcare facilities before reaching a public healthcare facility (57.9%) for definitive stroke care, often with or without a referral, due to financial constraints. The median time taken to reach a stroke care facility was 6.75 hours (IQR: 2.27-17.48), and the median distance from home to the final stroke care facility was 18.05 km (IQR: 8.175-35.792). The median hospitalization duration was five days (IQR: 4-9).

The median mRS score at admission was 4 (moderately severe disability) (IQR: 3-5), at discharge was 3 (moderate disability) (IQR: 2-5), and at the time of the survey was 2 (slight disability) (IQR: 2-3). The median EQ-5D-5L utility score was 0.78597 (IQR: 0.50068-0.9323), suggesting a reasonably good health state. More than 60% of participants (n = 113, 66.5%) were independent in performing activities of daily living (ADL), while 57 (33.5%) required assistance. However, more than 70% (n = 126, 74.1%) reported experiencing psychosocial issues post-stroke. Additionally, the median EQ-Health Analog Scale score was 60 (IQR: 40-75), indicating that while participants perceived their health as better, it was not at an optimal level (Table 1).

Prehospital delay and its correlates

Among the 170 stroke patients, 68 (40%) arrived at a stroke care facility more than four and a half hours after symptom onset. A majority (65.8%) sought care at multiple facilities, often starting at small private clinics or hospitals before eventually reaching acute or comprehensive stroke care centers, with the number of facilities visited ranging from two to four.

Bivariate analysis using the Chi-square test identified a statistically significant association between prehospital delay and factors such as age, income source, occupation, socioeconomic status, choice of healthcare facilities, and the presence of dependents (p < 0.05). Patients who arrived late were more likely to be employed, belong to the APL category, and have dependents (p < 0.05) (Table 2).

**Table 2:** Correlates of prehospital delays ^*^ p < 0.05 indicates statistical significance based on the Chi-square test. APL, above poverty line; BPL, below poverty line; mRS, Modified Rankin Scale

Variables		Early arrival, N (%)	Late arrival, N (%)	OR	p-Value
Gender	Male	67 (39.4%)	46 (27.1%)	0.916	0.791
	Female	35 (20.6%)	22 (12.9%)		
Type of residence	Rural	73 (42.9%)	54 (31.8%)	0.653	0.249
	Urban	29 (17.1%)	14 (8.2%)		
Type of house	Kaccha or semi-kachha	26 (15.3%)	31 (18.2%)	0.408	<0.05^*^
	Pucca	76 (44.7%)	37 (21.8%)		
Marital status	Married, living with spouse	77 (45.3%)	59 (34.7%)	0.47	0.072
	Never married/married, not living with spouse	25 (14.7%)	9 (5.3%)		
Education	Primary education to higher secondary	83 (48.8%)	52 (30.6%)	1.344	0.439
	Diploma to postgraduate or above	19 (11.2%)	16 (9.4%)		
Presence of income	No	28 (16.5%)	19 (11.2%)	0.976	0.944
	Yes	74 (43.5%)	49 (39.8%)		
Income source	Pension	53 (31.2%)	23 (13.5%)	0.89	<0.05^*^
	Salary	19 (11.2%)	20 (11.8%)		
	Others	2 (1.2%)	6 (3.5%)		
	Not applicable	28 (16.5%)	19 (11.2%)		
Occupation	Employed	51 (30%)	45 (26.5%)	0.511	<0.05^*^
	Unemployed	51 (30%)	23 (13.5%)		
Socioeconomic status	BPL	31 (18.2%)	33 (19.4%)	0.463	<0.05^*^
	APL	71 (41.8%)	35 (20.6%)		
Household head	Patient	42 (24.7%)	34 (20%)	0.7	0.257
	Others	60 (35.3%)	34 (20%)		
Decision-maker in household	Patient	52 (30.6%)	29 (17.1%)	0.873	0.527
	Joint including patient	5 (2.9%)	3 (1.8%)		
	Others	45 (44.1%)	36 (52.9%)		
Presence of multiple comorbidities	No	32 (18.8%)	28 (16.5%)	0.653	0.19
	Yes	70 (41.2%)	40 (23.5%)		
Type of facility opted for final stroke care	Private	50 (29.4%)	21 (12.4%)	2.152	<0.05^*^
	Public	52 (30.6%)	47 (27.6%)		
Stroke type	Ischemic	95 (55.9%)	61 (35.9%)	0.24	0.627
	Hemorrhagic	3 (1.8%)	4 (2.4%)		
	Others	4 (2.4%)	3 (1.8%)		
Health insurance	Yes	72 (42.6%)	40 (23.7%)	1.738	0.093
	No	29 (17.2%)	28 (16.6%)		
mRs during admission	Good outcome	22 (12.9%)	6 (3.5%)	2.842	<0.05^*^
	Bad outcome	80 (47.1%)	62 (36.5%)		
mRs at present	Good outcome	67 (39.9%)	38 (22.6%)	1.548	0.144
	Bad outcome	33 (19.6%)	30 (17.9%)		

**Figure 1:**
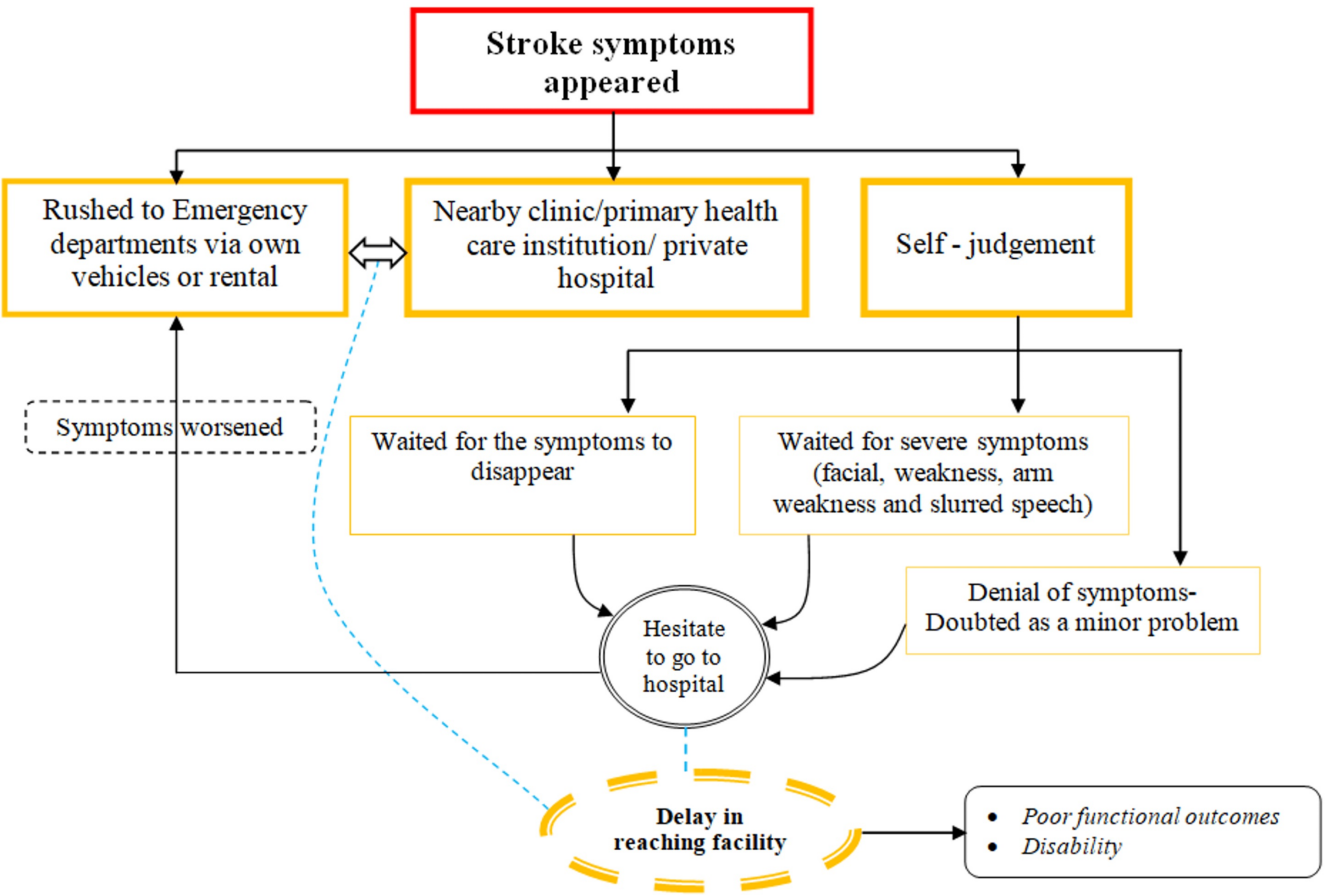
Developed framework

The freewheeling interviews revealed that, regardless of sociodemographic characteristics, hesitation, reluctance, and self-judgment were the primary causes of delay. Many patients and their families perceived certain symptoms - such as headaches, diarrhea, nausea, and palpitations - as minor and therefore ignored them. The belief that regularly taking medications for noncommunicable diseases would prevent a stroke, combined with the perception of being generally healthy, further delayed their decision to seek medical attention. Their understanding of typical stroke symptoms, including limb weakness, paralysis, or facial drooping, led them to underestimate the severity of their condition. This misjudgment resulted in delayed medical care, allowing symptoms to worsen. In some cases, particularly among women and young men, individuals waited for additional symptoms to appear before seeking medical attention (Figure 1).

*Around 10:00 am, I experienced slight pain and numbness in my right palm. I tried massaging it with balm, but it didn’t help, so I went to XXX hospital for an orthopedic consultation. I received some medication and returned home. Around 1:00 pm, I started having balance issues, which I attributed to high blood pressure and the side effects of the medication. I ignored it and lay down for a while. Later, I went outside where my wife was washing clothes, and she insisted I go to the hospital, but I wasn’t interested. Suddenly, I collapsed, and my upper limb felt heavy. I was immediately taken to a private hospital, where the doctors informed me that I had arrived too late* - 54-year-old male

*Morning. I was preparing tea for everyone at 6:30 am when I started feeling shivery and uncomfortable, but I didn't pay much attention because I was busy preparing lunch for my husband, who needed to go to the office, and my children, who had to go to school. I continued with my tasks, but later, I collapsed and lost consciousness. When I woke up, I was admitted to the hospital due to a stroke. I was puzzled about how this could have happened since my limbs weren’t paralyzed; I could sit and eat. How did this happen?* - 47-year-old female

The study found that participants who sought stroke care at public healthcare facilities experienced delays at twice the rate of those who went to private hospitals, with a statistically significant association (p < 0.05). This trend was also evident in freewheeling interviews. Patients and their families often face confusion and uncertainty about symptoms, making it more difficult to decide where to seek treatment. A common pattern emerged: many patients first visited small local clinics, then moved on to private hospitals, only to be referred to public hospitals later - by which time their symptoms had worsened. This delay was often attributed to inadequate infrastructure at smaller facilities or the inability to afford private care, as reported by patients and their families.

*Early morning, around 6.00 am, I felt so tired and I vomited. And at that time urine and motion also went. After the onset of symptoms at 6:00 am, I went to a XXX clinic near my home by 8.00 am. My son and daughter-in-law were with me. Their presence facilitated a quick response, although initially, they were unsure where to go. I told them to take me to that clinic as I regularly visit it for my health issues. Then I got medicines and reached home. After one hour, I vomited and felt so tired and lost my balance (‘vaadippoyi’) then we went to XXX hospital (private) at 9.45 am then they referred me to XXXX (public) at 11:00 pm due to inadequate facilities for stroke care* - 55-year-old male stroke survivor

**Figure 2:**
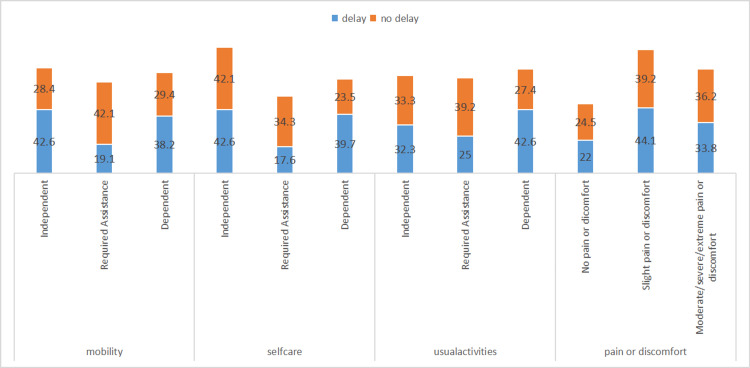
Delay and functional measures

Furthermore, a significant association was observed between prehospital delay and the mRS score at admission (p = 0.028), with delayed patients tending to have worse initial outcomes. However, the current mRS status does not show a statistically significant association with delays (p = 0.144), although poorer outcomes remain more common among those who experienced delays (Table 2). Additionally, stroke patients who faced prehospital delays generally reported lower quality of life outcomes compared to those who reached the hospital in a timely manner (Figure 2).

Logistic regression analysis revealed that socioeconomic factors, including housing type, indecision in selecting a hospital for stroke care, and the number of facilities considered for hospitalization, significantly increased the likelihood of delays (p < 0.01). Patients who sought treatment at public hospitals were 1.018 times more likely to experience delays compared to those who received care at private hospitals. Older patients showed a slightly lower tendency for delays. Although unemployed individuals and those with dependents experienced more delays, these associations were not statistically significant (Table 3).

**Table 3:** Regression analysis ^* ^p < 0.01 indicates statistical significance. APL, above poverty line; BPL, below poverty line

Variable	Categories	OR	p-Value
Duration of stroke	≤6 months (reference category)		0.068
	>6 months	0.391	
Facility opted for stroke care	Private (reference category)		0.972
	Public	1.018	
Occupation	Employed (reference category)		0.239
	Unemployed	1.878	
Socioeconomic status	BPL (reference category)		0.361
	APL	1.604	
Income source	Pension and old age pension (reference category)		0.688
	Salary	1.276	0.713
	Not applicable	1.291	0.706
	Others	4.028	0.225
Type of house	Kachha or semi-kachha (reference category)		0.038
	Pucca	2.958	
Age		0.986	0.575
Number of facilities opted for hospitalization		17.665	p < 0.01^*^
Number of dependents		1.313	0.074

## Discussion

Stroke and its associated disabilities are recognized as a major global public health concern, significantly contributing to the overall disease burden [15-17]. Despite advancements in stroke management, many acute stroke cases do not receive timely medical attention, even in high-income countries [18]. Thrombolytic treatment is most effective within a therapeutic window of less than four and a half hours, as recommended by the AHA, with the best outcomes observed when administered within 90 minutes. However, due to significant prehospital delays, only a small number of acute stroke patients receive thrombolytic therapy [19,20].

This study examines prehospital delays and their associated factors among stroke patients admitted to both public and private hospitals in the Thiruvananthapuram district, Kerala. The median time taken by patients to reach a stroke care facility in our study was 6.75 hours, with an IQR of 2.27-17.48 hours. This is particularly concerning, given that timely medical intervention is essential for optimizing stroke outcomes. The delays observed in our study are consistent with findings from studies conducted in India, Nepal, China, the USA, Greece, and the UK [21-25]. These delays can be attributed to several factors, including a lack of public awareness about stroke symptoms, failure to recognize the urgency of the situation, and inadequate emergency response systems. Many patients and their families hesitate to seek help due to uncertainty about symptoms or logistical challenges, such as transportation difficulties and the time required to locate and reach an appropriate stroke care facility. This pattern was also evident in our study, as reflected in free-wheeling interviews with patients. Additionally, the median distance from home to the final stroke care facility was approximately 18 km.

Consistent with previous studies, our findings indicate an inverse relationship between age and prehospital delay in stroke patients, with older patients being less likely to experience delays. A significant association was found [26-28]. Younger patients, particularly those under 55, were more prone to hospital delays. They may not immediately recognize the severity of stroke symptoms, misinterpret them as less serious health issues, or underestimate the urgency required for treatment, leading to delays in reaching a healthcare facility. These age-related differences in health-seeking behavior underscore the importance of tailored strategies to address specific barriers faced by different age groups.

According to previous studies, lower socioeconomic status is linked to longer delays in seeking medical care. However, the present study found that individuals from higher socioeconomic classes experienced longer delays in reaching stroke care facilities [29-31]. Those from higher socioeconomic backgrounds often have greater access to private healthcare facilities, which may be located farther from their residence, increasing travel time and contributing to delays. Additionally, individuals with greater autonomy in decision-making may explore various treatment options or seek multiple opinions before deciding on a hospital, further delaying care. In contrast, individuals from lower socioeconomic backgrounds may have more direct pathways to care, often relying on local healthcare providers or government-run facilities, which can result in quicker access to medical treatment [32].

Contrary to the findings of Alkhotani et al., employed individuals in our study experienced more delays in reaching stroke care facilities [33]. Early symptoms such as headache, diarrhea, or vomiting were often unrecognized and mistakenly attributed to stress at work, leading to delays in seeking medical attention. The patient’s perception of symptom severity and their knowledge of stroke symptoms and management options directly influenced decision delays, contributing to prehospital delay. Previous studies have also reported similar relationships [34,35].

Unnecessary referrals frequently lead to delays in reaching hospitals and receiving timely care [33,36]. These delays can be attributed to a lack of infrastructure for managing acute stroke cases and insufficient awareness among patients and their families about nearby acute stroke care facilities. Furthermore, trust in the public healthcare system and the strong promotion of private healthcare options may encourage patients and their families to approach multiple facilities before receiving appropriate care.

A common trend observed in our study was that patients initially sought treatment at the nearest healthcare facilities, particularly small private clinics or primary healthcare institutions. These facilities were chosen for managing symptoms such as vomiting, headache, and diarrhea, which can be early signs of stroke. However, as symptoms worsened over time, patients were compelled to seek more advanced care at secondary or tertiary healthcare facilities. This delay in accessing appropriate care was further exacerbated when patients were referred from private hospitals to public healthcare facilities due to financial constraints. In many cases, patients initially opted for public hospitals, but due to inadequate infrastructure, significant delays in obtaining diagnostic reports (including CT scans), and a lack of available beds, family members themselves decided to transfer patients to another hospital. These challenges were prominently reflected in free-wheeling interviews with the family members of stroke patients, highlighting systemic issues within the public healthcare system that contribute to delays in care. This situation underscores the need for improved infrastructure and resource allocation in public healthcare facilities and raises critical concerns about equitable access to timely stroke care for all patients.

These findings emphasize the urgent need to strengthen the state government’s flagship program, the SIRAS initiative. This program aims to ensure timely acute stroke care by establishing primary stroke care units in district and general hospitals across Kerala, providing free thrombolysis treatment using alteplase/tenecteplase [36]. Patients who experienced delays in reaching the hospital exhibited higher levels of dependence in performing ADL and poorer quality of life [37-39]. This highlights the importance of rapid intervention and the need to address factors contributing to these delays, including logistical challenges, lack of awareness, and systemic inefficiencies within healthcare delivery. These insights reinforce the critical need for enhanced emergency response systems and the strengthening of public healthcare institutions through coherent policies, leadership, and governance.

Consistent with previous studies, patients who experienced delays in reaching the hospital were more likely to have worse mRs scores upon admission, with a significant association found [40]. Although no significant association was observed at discharge or in current mRs scores, worse outcomes remained more prevalent among those who faced delays. This underscores the importance of prompt interventions in acute stroke care facilities.

Overall, these findings highlight the need for a comprehensive, multipronged approach to address the socioeconomic and systemic factors contributing to prehospital delays in stroke care. The study recommends further research to explore the sociocultural and systemic barriers leading to prehospital and treatment delays among stroke patients. Such studies would be valuable in developing targeted strategies to improve stroke care outcomes across various healthcare settings.

Limitations

This study was conducted in a single district, which limits the generalizability of the findings to the broader Indian population. Patients who passed away before reaching the hospital were excluded. Since the study focused on prehospital factors, delays and treatments that occurred after hospital arrival were not captured. As a cross-sectional study, it cannot establish causality between the identified factors and prehospital delays. Recall bias is also a potential limitation, as stroke patients or their attendants may have inaccurately reported the time of symptom onset. However, this was mitigated by selecting the most reliable informant. Additionally, information bias may have arisen from self-reported comorbidities. To address this, each condition was inquired about in the local language, and responses were verified against discharge summaries. Current medications were also cross-checked for accuracy.

## Conclusions

This study highlights significant challenges in timely access to stroke care in Thiruvananthapuram, with 40% of stroke patients experiencing prehospital delays. The median delay of 6.75 hours is particularly concerning, given the critical importance of prompt intervention in reducing stroke-related mortality and disability. Factors such as age, socioeconomic status, occupation, and the presence of dependents were significantly associated with these delays, underscoring systemic barriers that need to be addressed. Additionally, psychological and cultural factors, including hesitation, self-judgment, and the underestimation of symptoms, further contributed to delayed hospital arrival. Improving stroke outcomes requires targeted educational initiatives to enhance public awareness of stroke symptoms and the urgency of seeking immediate care. Streamlining referral processes and ensuring rapid access to appropriate healthcare facilities are also essential steps in reducing prehospital delays and improving patient outcomes. A holistic approach to addressing these barriers will be key to reducing the burden of stroke and enhancing the quality of care.
